# Evaluation of mast cell counts and microvessel density in reactive lesions of the oral cavity

**DOI:** 10.15171/joddd.2016.038

**Published:** 2016-12-21

**Authors:** Maryam Kouhsoltani, Monir Moradzadeh Khiavi, Shabnam Tahamtan

**Affiliations:** ^1^Dental and Periodontal Research Center and Faculty of Dentistry, Tabriz University of Medical Sciences, Tabriz, Iran; ^2^Department of Oral and Maxillofacial Pathology, Faculty of Dentistry, Tehran University of Medical Sciences, Tehran, Iran; ^3^Postgraduate Student, Biotechnology Research Center and Department of Orthodontics, Faculty of Dentistry, Tabriz University of Medical Sciences, Tabriz, Iran

**Keywords:** CD31, lesion, mast cell tryptase, oral cavity, reactive

## Abstract

***Background.*** Reliable immunohistochemical assays to assess the definitive role of mast cells (MCs) and angiogenesis in the pathogenesis of oral reactive lesions are generally not available. The aim of the present study was to evaluate mast cell counts (MCC) and microvessel density (MVD) in oral reactive lesions and determine the correlation between MCC and MVD.

***Methods.*** Seventy-five cases of reactive lesions of the oral cavity, including pyogenic granuloma, fibroma, peripheral giant cell granuloma, inflammatory fibrous hyperplasia, peripheral ossifying fibroma (15 for each category) were immunohisto-chemically stained with MC tryptase and CD31. Fifteen cases of normal gingival tissue were considered as the control group. The mean MCC and MVD in superficial and deep connective tissues were assessed and total MCC and MVD was computed for each lesion.

***Results***. Statistically significant differences were observed in MCC and MVD between the study groups (P < 0.001). MC tryptase and CD31 expression increased in the superficial connective tissue of each lesion in comparison to the deep con-nective tissue. A significant negative correlation was not found between MCC and MVD in oral reactive lesions (P < 0.001, r = -0.458).

***Conclusion.*** Although MCs were present in the reactive lesions of the oral cavity, a direct correlation between MCC and MVD was not found in these lesions. Therefore, a significant interaction between MCs and endothelial cells and an active role for MCs in the growth of oral reactive lesions was not found in this study.

## Introduction

MCs are granule-containing bone marrow-derived immune cells.^1^These cells are derived from the multipotent CD34 precursors in the marrow and circulate in the blood as monocytes.^2^ Immature MCs obtain their characteristic granular morphology after migrating into tissues.^3^ Mature MCs are spread throughout tissues, including connective tissues and mucosal environments.^4^ These cells play a role in hypersensitivity reactions, inflammatory processes, connective tissue remodeling and wound healing.^1,5^

It is not certain whether MCs promote the development or suppress the development of lesions. MCs exhibit a significant participation in angiogenesis by producing numerous elements, including tryptase, FGF, IL-8, VEGF, heparin, TNF and histamine. Neo-vascularization is the development of new blood vessels from the progenitors of endothelial cells or pre-existing vessels. This phenomenon, by providing oxygen and nutritional substances, has a critical role in the growth of the lesions and tumor metastasis.^5-8^ Many researchers have investigated the role of MCs in vasoinductive events and MCs have been shown to promote neo-angiogenesis in various lesions.^5-7^ Moreover, MCs may confine development of the lesions by producing numerous elements such as TNF and IL-1, IL-4 and IL-6. In addition, MC granules exert effects on fibroblasts, and fibrosis may have a role in limiting tumor growth.^6,9^

The definitive role of MCs in the growth of oral reactive lesions is an issue of debate. To date, limited studies have been carried out to investigate the role of these cells in oral reactive lesions and very little has been found about angiogenesis in these lesions. Reactive lesions of the oral cavity are hyperplasic reactions of the connective tissue elements when exposed to low-grade and chronic trauma or regional irritation. These are the most common lesions in the oral cavity^1,10-11^Distribution of MCs in oral reactive lesions was investigated in a study by Farahani *et al,*^1^ in which MCCs in the reactive lesions of the oral cavity were compared with normal gingival tissue. Sudhakar et al^3^ evaluated MCs in the inflammatory lesions of the oral cavity. Farahani et al used toluidine blue to stain oral reactive lesions, including peripheral giant cell granuloma (PGCG), fibroma (F), peripheral ossifying fibroma (POF) and inflammatory fibrous hyperplasia (IFH), and also normal gingival tissue. They reported that MC counts were higher in reactive lesions of the oral cavity in comparison to normal gingival tissue. Sudhakar et al also used toluidine blue to stain MCs in inflammatory oral lesions (three PGs, six IFHs, five granulation tissues, and one gingivitis case). They also evaluated the relation between MC counts and vascularity in the sections stained with toluidine blue, and did not use a special marker to evaluate vascularity. They found a negative relation between MC counts and vascularity.

This study was thus carried out to count MCs and microvessels in the reactive lesions of the oral cavity. Our aim was to assess the role of MCs in the growth of oral reactive lesions, which might help to improve knowledge about the pathogenesis of these lesions for therapeutic purposes.

## Materials and Methods

In this cross-sectional study, 75 formalin-fixed, paraffin-embedded tissue blocks were taken from the laboratory archives of the Department of Oral and Maxillofacial Pathology, Tabriz University of Medical Sciences, from 2003 to 2013. Five study groups (15 samples for each category) were considered in this work, including inflammatory fibrous hyperplasia (IFH), fibroma (F), peripheral ossifying fibroma (POF), peripheral giant cell granuloma (PGCG), and pyogenic granuloma (PG). All the cases were assessed to have sufficient clinical records and all the slides were reviewed to confirm the diagnosis. The selection criteria included the presence of an intact epithelium, extension of at least 10 high-power fields (HPFs) for each sample, and a proper paraffin block for preparing new slides. The control group was composed of fifteen samples of normal gingival tissue acquired from patients who had undergone surgery for impacted third molars.

Two 4-μm sections were cut from formalin-fixed and paraffin-embedded tissue blocks. Staining of the cut sections was carried out following standard immunohistochemical guidelines (DAKO, Glostrup, Denmark)‏. The sections were placed onto slides and then deparafinized in xylene and rehydrated in alcohol. Hydrogen peroxide (1%) was applied to occlude endogenous peroxidase activity. In order to retrieve antigens, the slides were immersed in citrate buffer solution (pH = 6.0, 0.01 M) for 20 minutes. The samples were then incubated with 1:20 anti-human mouse primary antibodies for CD31 and 1:100 diluted antibodies for MC tryptase, and afterwards incubated with secondary antibody; 0.3% diaminobenzidine (Dako Cytomation) solution was applied to detect secondary antibodies.

Analysis included observing the slides at a magnification of ×100 s and detecting the areas most populated by MCs and microvessels (hot spot fields). Two observers assessed ten HPFs (high-power fields), with an agreement of 92%. Five of ten HPFs represented MCCs and MVDs in subepithelial connective tissues, and five remaining HPFs represented MCCs and MVDs in deep connective tissues. The means of the five HPFs were taken as the subepithelial and deep connective tissue MCCs and MVDs. The total means of the ten HPFs were considered as the total MCC and MVD for each sample.

Statistical analysis was carried out using SPSS 20.0 (SPSS, Chicago, IL). Interobserver reproducibility for both MCC and MVD was determined by six double assessments for each. Mean ± standard deviation (SD) was calculated for each group. One-way ANOVA followed by HSD Tukey test was carried out for comparison of MCCs and MVDs amongst groups. In each lesion, MCCs and MVDs in the depth of connective tissue and under the epithelial surface were compared using paired t-test. To evaluate the correlation between MCC and MVD, Spearman’s rank correlation coefficient was used. Significance was established at a P < 0.05.

## Results

### Clinical findings

Complete records and biopsy materials from 75 cases of oral reactive lesions (15 samples for each category) and 15 samples of normal gingiva were assessed in this study. Specimens included the following categories: F [(7 females (F) and 8 males (M)]; 20 to 66 (mean 44.4) yrs.); PGCG [(6 F and 9 M; 6 to 51 (mean 32) yrs.]; IFH [(13 F and 2 M; 14 to 66 (mean 49.6) yrs.)]; POF [(6 F and 9 M; 14 to 42 (mean 25) yrs.]; and PG [(9 F and 6 M; 13 to 68 (mean 43.7) yrs.]. Totally, 41 out of 75 cases (54%) occurred in females. This finding indicated that sex distribution was relatively similar in the studied lesions. Seven cases of F (46.6%), 9 cases of POF (60%), 7 cases of IFH (46.7%), 8 cases of PG (53.3%) and 7 cases of PGCG (46.7%) were located in the maxilla. These results showed that POF and PG occurred more commonly in the maxilla.

### Immunohistochemical findings

**Figure 1.**
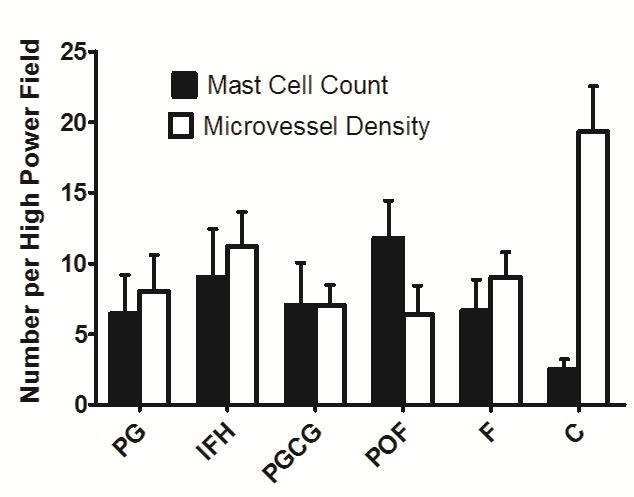
Tryptase-positive mast cells in: a) control group, b) fibroma, c) inflammatory fibrous hyperplasia, d) peripheral ossifying fibroma, e) pyogenic granuloma, f) peripheral giant cell granuloma (IHC staining, ×400).

**Figure 2.**
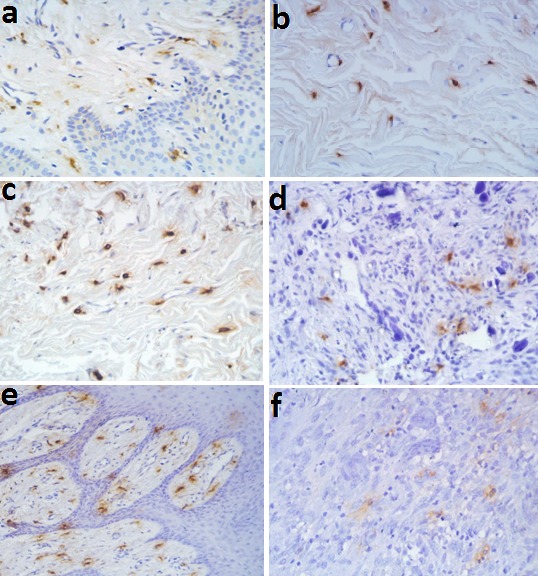
Histograms of mast cell counts and microvessel densities in oral reactive lesions.

In microscopic sections, MCs were frequently, located near the blood vessels, generally in the lamina propria. The mean MCCs in the studied groups are illustrated in Table 1, Figure 1, and Figure 2. One-way ANOVA showed statistically significant differences in MCCs between the study groups (P < 0.01). The level of significance was considered at 5%

**Table 1:** Mast cell counts and microvessel densities in oral reactive lesions

**Study groups**	**Number of cases**	**Mast cell counts**		**Microvessel densities**	
		P-value^*^	P-value^*^	**SD± Mean**	**SD± Mean**
**PG**	15	<0.001	<0.001	0.37‏ ± 2.90	6.43 ± 2.75
**IFH**	15			11.19 ± 2.45	9.04 ± 3.38
**PGCG**	15			7.03 ± 1.45	7.03 ± 3.01
**POF**	15			6.42 ± 2.00	11.78± 2.66
**F**	15			9.03 ± 1.77	6.63 ± 2.22
**C**	15			19.32 ± 3.20	2.51 ±0.70

^*^One-way ANOVA F: fibroma, IFH: inflammatory fibrous hyperplasia, PGCG: peripheral giant cell granuloma, POF: peripheral ossifying fibroma, PG: pyogenic granuloma

**Figure 3.**
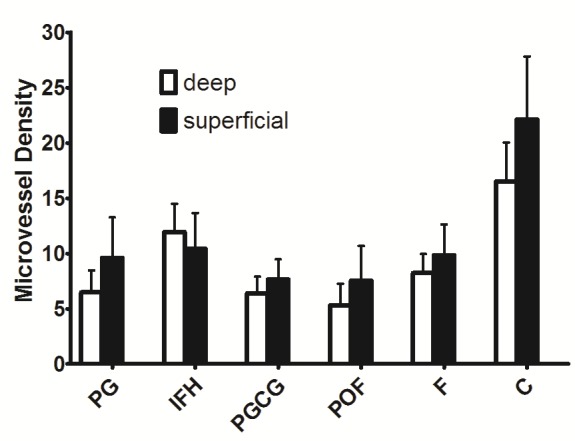
CD31-positive microvessels in: a) control group, b) fibroma, c) inflammatory fibrous hyperplasia, d) peripheral ossifying fibroma, e) pyogenic granuloma, f) peripheral giant cell granuloma (IHC staining, ×400).

The mean MVD in oral reactive lesions is illustrated in Figure 2, Figure 3 and Table 1. A significant difference in MVDs between the groups was shown by one-way ANOVA at the 5% level of significance (P < 0.01).

**Figure 4.**
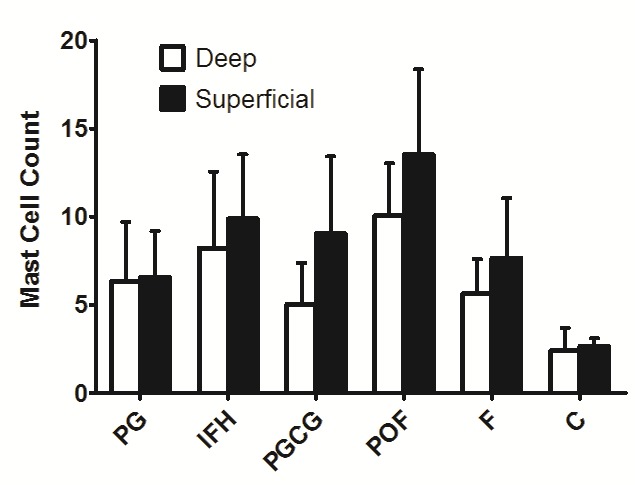
Mast cell counts in superficial and deep connective tissues in oral reactive lesions.

**Figure 5.**
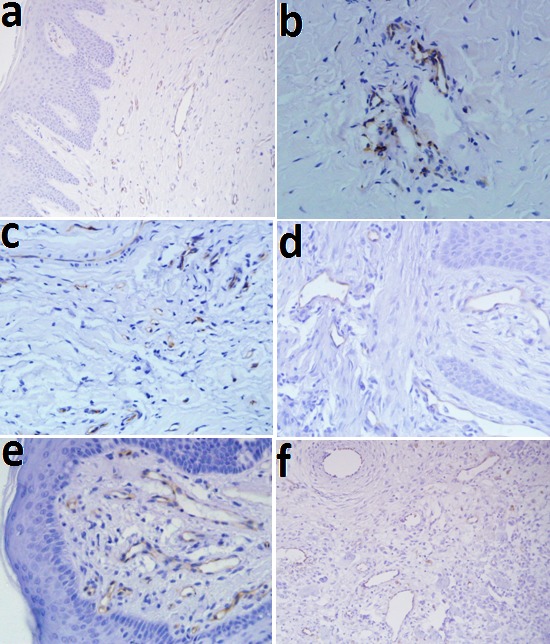
Microvessel densities in superficial and deep connective tissues in oral reactive lesions.

**Figure 6.**
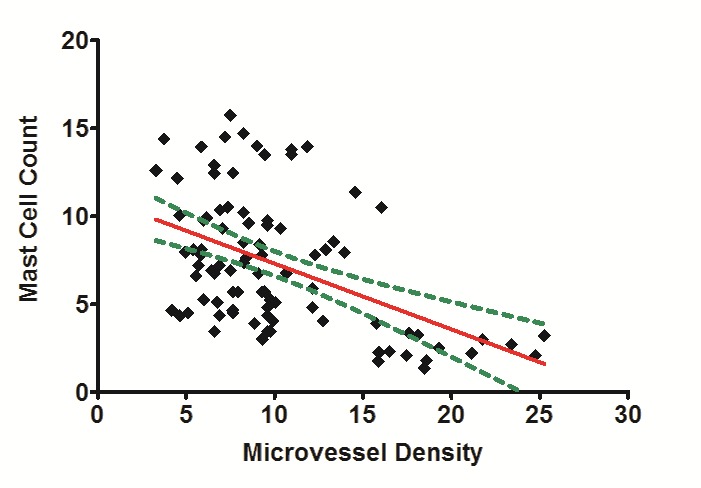
Correlation between mast cell counts and microvessel density in oral reactive lesions.

MC and CD31 expressions increased under the epithelial surfaces compared to the depth of the connective tissues (Figures 4 and 5). Paired t-test showed that these increases were statistically significant in POF, PGCG and F groups for MC tryptase expression and in PG, PGCG, F and control groups for CD31 expression (P < 0.05). A significant and negative correlation was found between MCC and MVD using Spearman's rank correlation coefficient (Figure 6) (P < 0.001, r = -0.458).

## Discussion

Tryptase is a serine protease that is secreted by activated MCs, leading to high levels in plasma and other body fluids.^5^ We specifically utilized tryptase for staining the sections because it identifies intact and degranulated MCs with high degree of sensitivity and precision. Tryptase is detected in human MCs, but is not found in the other cell types. Therefore, this marker is the most reliable indicators of MCs.

Tryptase is implicated in various biologic activities, e.g. induction of the proliferation of smooth muscle cells and fibroblasts.^4^ This protein directly stimulates the proliferation and migration of endothelial cells or indirectly degrades the connective tissue matrix, thus making space for neo-vascularization. Furthermore, MCs are a source of vascular endothelial growth factor (VEGF).^5,12^ In this study, we selected CD31 to assess angiogenesis. CD31, also recognized as platelet endothelial cell adhesion molecule-1 (PECAM-1), is an important factor for adhesion between endothelial cells that occurs during angiogenesis,^13^ and CD31 has emerged as one of the most valuable markers of endothelial cells.^14^

In this study, first we comprehensively assessed MC tryptase expression in reactive lesions of the oral cavity. MCCs were higher in all the lesions in comparison to the normal gingiva. This finding is consistent with the results of studies by Reddy et al,^2^ Farahani *et al,*^1^ and Sudhakar et al.^3^ Other studies have also demonstrated higher of MC counts in oral PG, giant cell fibroma and IFH (30 samples of PG were evaluated using toluidine blue, and 30 cases of giant cell fibroma and 30 cases of IFH were assessed using anti-tryptase antibody).^4,15^Our findings revealed that MC tryptase expression was higher in POF and IFH, consistent with the results of a study by Farahani *et al.*^1^ They suggested that since IFH and POF have denser fibrotic stroma, MCs might play a role in collagen synthesis in these lesions. Reddy et al.^2^ showed that MCCs were increased in F and POF that probably demonstrates the role of MCs in fibrous changes of the stroma in oral reactive lesions.

Our findings revealed that the distribution of MCs across all zones of the reactive lesions were not the same. MC tryptase expression was higher under the epithelial surface in comparison to the depth of connective tissue. These results suggest that MCs do not have a significant role in the growth of these lesions. This kind of comparison has not been made before, except for a study by Santos et al,^4^ in which immunoreactivity to MC tryptase antibody was evaluated in the epithelium and the connective tissue of IFH and giant cell fibroma. However, these two fields were not compared in this study. This finding is comparable with the localization of MCs in odontogenic cysts in subepithelial regions.^16-17^

In the second part of the present work, we assessed CD31 expression in oral reactive lesions and normal gingiva. According to our results, CD31 expression was higher in F and IFH groups.‏ Previous studies have not made such a comparison in oral reactive lesions. Our findings revealed that the distribution of microvessels across all the zones of reactive lesions were not the same. We observed higher MVDs under the epithelial surfaces in comparison to the depth of connective tissues. Seifi et al^18^ in a study of 15 follicular cysts, 15 odontogenic keratocysts and 15 ameloblastomas, similarly showed higher MVDs under epitheliums compared to the depth of connective tissues.

Our immunohistochemical studies did not demonstrate a significant and positive correlation between MCC and MVD. Studies evaluating the correlation between MCC and MVD have been performed by many authors in various lesions throughout the body.^5^ For example, a positive correlation between MCC and MVD was not found in lung cancer samples (non-small cell type)by Niczyporuk et al.^12^ On the other hand, Mohtasham et al^6^ found a direct correlation between MCC and MVD in oral dysplasia and squamous cell carcinoma samples and concluded that MCs promote tumor progression by upregulation of neo-vascularization. Soucek et al showed that MCs play a role in the expansion and angiogenesis of tumors of pancreas (islet cell tumors).^19^ Feoktistov et al reported that MCs stimulate angiogenesis through interaction between adenosine receptors (A3 and A2B).^20^ The correlation between MCC and MVD in oral reactive lesions was partly evaluated by Sudhakar et al^3^ and Reddy et al.^2^ In both studies, the correlations were assessed in sections stained with toluidine blue, and no specific markers for endothelial cells were applied. In the study by Sudhakar et al^3^ on 3 samples of PGs, 6 samples of IFHs, 5 samples of granulation tissues, and one sample of gingivitis^3^, and by Reddy et al on 40 samples of oral reactive lesions^2^, negative relations were found between MCs and vascularity.

Although there are clear data available about the stimulation of angiogenesis by MCs, it seems there is not always a direct relation between MVD and MCC in reactive lesions of the oral cavity. This may be because of the distinct pathobiologic and histopathological features of the different lesions in various sites of the body and due to the fact that different molecular pathways are involved in the process of development of various lesions. There is evidence that MCs individually or synergistically promote angiogenesis by producing several factors, including extracellular matrix-degrading enzymes (e.g. matrix metalloproteinase, tryptase, and chymase), TNF-alpha, VEGF, bFGF, IL-8, histamine and heparin. Furthermore, MCs induce non-endothelial cells through chemotactic stimulants. These neighboring cells synthesize several factors that promote or suppress angiogenesis, depending on the microenvironmental environments. As a result of different microenvironmental circumstances in various lesions, the local MCs show substantial modifications in function, phenotype and number. Therefore, comprehensive knowledge of MC-induced angiogenesis requires an understanding of the combination of receptors expressed by MCs and potential target cells (of MCs) and the complex relations between the factors affecting angiogenesis.^5-7^

## Conclusion

Although MCs were present in oral reactive lesions, a direct correlation between MCC and MVD was not found. Therefore, a significant interaction between MCs and endothelial cells and an active role for MCs in the growth of oral reactive lesions was not found in this study. Our findings demonstrated increased MCC in POF and IFH and thus, MC activation may have a role in the stimulation of fibrosis in some oral reactive lesions. Numerous interactions between MCs and different cell types can provide a basis for further therapies to target MC responses.

## Acknowledgements

This manuscript was obtained from thesis No. 1423, Faculty of Dentistry, Tabriz University of Medical Sciences and supported by Research Council of the same university.

## Authors’ contributions

MK participated in study design, literature search, data acquisition, data analysis and manuscript preparation. ST participated in literature search, data acquisition, data analysis and manuscript preparation. MM performed the paraclinical studies and participated in data acquisition and manuscript preparation. All the authors have read and approved the final manuscript.

## Funding

This manuscript was supported by Research Council of Tabriz University of Medical Sciences

## Competing interests

The authors declare no competing interests with regards to the authorship and/or publication of this article.

## Ethical approval

The research protocol was approved by the Ethics Committee of Tabriz University of Medical Sciences (Ref. No. 432).
